# The offonome reveals *on* and *off* states of gene expression near the detection limit of RNA-seq

**DOI:** 10.1038/s41598-025-27185-5

**Published:** 2025-11-28

**Authors:** Won-Young Choi, Xiaobei Zhao, Jeremiah Holt, Richa Jain, Heejoon Jo, Katherine A. Hoadley, D. Neil Hayes, Hyo Young Choi

**Affiliations:** 1 UTHSC Center for Cancer Research, University of Tennessee Health Science Center, Memphis, TN USA; 2 Methodist Le Bonheur Healthcare, Memphis, TN USA; 3 Department of Genetics, Lineberger Comprehensive Cancer Center, University of North Carolina at Chapel Hill, Chapel Hill, NC USA; 4 Department of Medicine, University of Tennessee Health Science Center, Memphis, TN USA; 5 Department of Preventive Medicine, University of Tennessee Health Science Center, Memphis, TN USA; 6 Current address: Department of Pathology, University of Tennessee Health Science Center, Memphis, TN USA

**Keywords:** High dimensional representation of gene expression, Low expressed genes, Bulk RNA-seq, Head and neck squamous cell carcinoma, Lung cancer, Computational biology and bioinformatics, Genome informatics

## Abstract

RNA-seq, widely used for gene expression profiling, provides nucleotide level genome coverage and summary gene expression values. Generally, low-expressed genes are ignored due to their unfavorable signal-to-noise ratio, however, these genes may offer crucial information, such as detecting rare cells in bulk tissues. In this study, we applied an approach that transforms the expression levels of low-expressed genes into a robust dichotomized *on*/*off* state by leveraging similarities in transcript coverage shape. Applied to three human cancer cohorts from the Cancer Genome Atlas (TCGA), chosen based on tissue morphology and anatomic site, we identified genes, the “offonome” near the detection limit, consistently or occasionally *off* across samples. Genes in the offonome spectrum proved useful for supervised and unsupervised applications, including characterizing oncogenic pathways, and identifying rare populations of cells in bulk tissue. Interrogating the offonome is relevant to bulk tumor analyses like TCGA, potentially expediting gene investigation in low-input situations like single cell RNA-seq.

**Supplementary Information:**

The online version contains supplementary material available at 10.1038/s41598-025-27185-5.

## Introduction

Over the last decade, short read RNA-seq has enabled a wide range of gene expression analysis including detection of differentially expressed genes, tumor subtypes identification/classification, deconvolution of gene expression, and gene network analysis^1–6^. In general, RNA-seq analysis software, such as DESeq2, edgeR and limma implement their own filtering and normalization procedures prior to analysis to correct for library sizes, technical biases, or unfavorable properties of genes^4,5,7^. A variety of empiric techniques have been employed to emphasize sets of highly expressed genes with favorable variance properties or exclude genes below a certain threshold of low expression. However, detailed considerations of genes expressed at low levels have not been extensively reported. Although removal of genes based on low signal might be desirable for the reasons stated above, we consider some of the disadvantages. Genes expressed near the limit of detection might nonetheless have important biologic activity in some but not all cells, such as stem cell populations in a differentiating tissue. Low gene expression may reflect biological activity of minor cell populations within bulk tissue sections, such as infiltrating inflammatory cells or minor populations of normal epithelial or stromal cells^1,8^. Deconvolution methods attempt to recover such populations without considering that filtering may have removed low levels of expression highly characteristic of minor cell populations.

As lowly expressed genes are typically defined based on their overall read counts across the entire cohort or by whether they fail to exceed a certain threshold in a defined proportion of samples, genes that are “truly” expressed in a small subset of samples sometimes are overlooked. Our group has recently reported a high dimensional representation of gene expression which potentially augments the signal to noise ratio such that removal of many lowly expressed genes might not be required^9,10^. In the current report, we demonstrate how alternative transformations of short read RNA-seq can be utilized to robustly use a part of the transcriptome which has largely been removed from consideration. In the extreme case, we offer a definition that characterizes genes as *off* or *on* (the offonome) and demonstrates the ability to detect meaningful biological signals that are difficult to detect using only count-based information. By our definition, offonome mainly includes genes that have low read counts in overall cohort. At the same time, if genes have *on* status even in a very small subset of cohort, they are also included regardless of their average expression level.

By using offonome, we apply our previously described high-dimensional representation of RNA expression called level of shape similarity (LSS) to three tumor types selected to represent the shared boundaries of two anatomic sites (lung versus head and neck) and two morphologic cancer cell types (squamous cell carcinoma versus adenocarcinoma). Our study demonstrates that the offonome effectively detects different normal cell infiltration based on tumor sites and proves its potential application in characterizing each cancer type, revealing distinct clusters and associated gene ontologies. Furthermore, the offonome exhibits the capability to distinguish three tumor types, even comprised of low-expressed genes highlighting its utility for investigating genes at the lower end of the expression spectrum which can augment gene sets defined by highly expressed genes.

## Results

The study encompassed all available TCGA tumor samples (N = 1509) with RNA-seq data from three cancer types: Head and Neck Squamous Cell Carcinoma (HNSC; N = 514), Lung Squamous Cell Carcinoma (LUSC; N = 486) and Lung Adenocarcinoma (LUAD; N = 509). Two of the three datasets share squamous cell carcinoma morphology and the third adenocarcinoma morphology, such that genes associated with squamous morphology versus adenocarcinoma could be assessed in two datasets as an internal control. Likewise, two of the datasets originated within the anatomic site of the lung, such that genes associated with lung anatomic site versus head and neck sites could be investigated with an internal validation control. By filtering out severely degraded samples, we identified 1,252 usable specimens from the TCGA repository which have diverse clinical stages: 418 HNSC, 419 LUAD and 415 LUSC (Supplementary Table 1 and Supplementary Table 2, See Methods).

### High-dimensional shape-based approach to reliably measure low gene expression

**Fig. 1:**
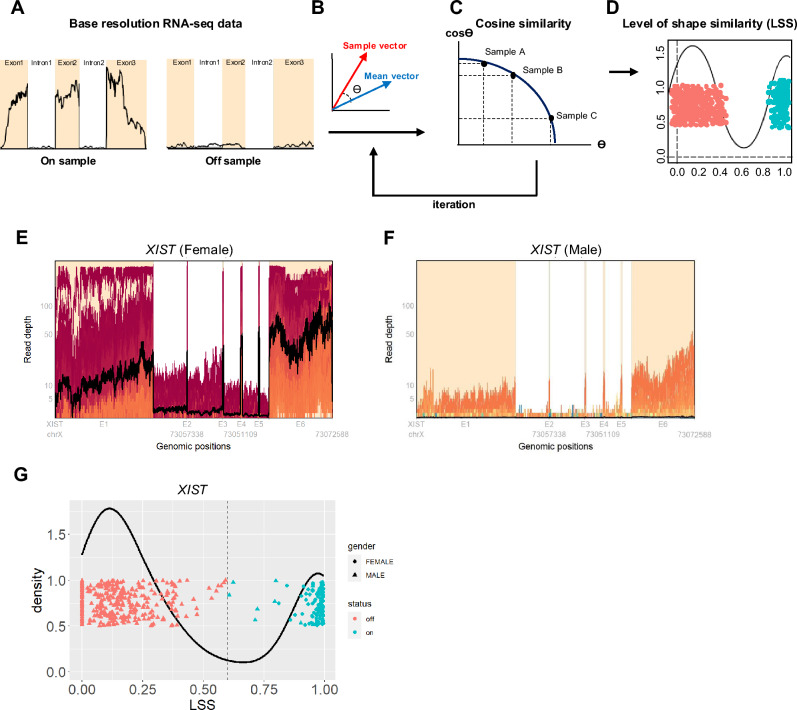
Characterizing *on* and *off* status in gene expression by using base resolution RNA-seq data. The process of estimating LSS values. **a** Collecting the single base resolution RNA-seq data. **b** After transforming base resolution pileup data to high dimensional vector, calculating angle between mean vector and sample vector. **c** Based on the angle between sample and mean vector, cosine similarity values can be derived. These steps are iterated until the value is optimized. **d** Final cosine similarities can be described by the level of shape similarity (LSS). **e** Single base resolution RNA-seq coverage plot of *XIST* gene for females. **f** Single base resolution RNA-seq coverage plot of *XIST* gene for males. Both sample data were derived from HNSC cohort. Orange boxes annotated by E1–E6 in x-axis indicate exon regions and white boxes indicate intron regions. Y-axis indicates read depth of single base location. Black line in pileup data shows the mean value of all samples involved in each pileup panel. **g** Density plot of LSS value of *XIST* for HNSC cohort. To divide the samples of gender, we used circle indicating females and triangle indicating males. Also, vermilion color indicates samples that have off status for the *XIST* gene and sky blue indicates samples that have on status for the *XIST* gene. Dotted line indicates 0.6 of LSS. To avoid the overlap, we intentionally separated each sample dot by adding random scatter in the Y-axis.

We used the established approach, Level of Shape Similarity (LSS), to determine per-sample gene expression status (*on*/*off*) from transcript-coverage shape. LSS compares each sample’s base-resolution coverage vector with a cohort-wide mean profile, capturing the inherently structured and non-uniform RNA-seq coverage. Transcript coverage reflected transcript architecture (exons and introns), while also accommodating additional variation arising from technical or biological factors including GC content and RNA integrity^9,11–13^ (Fig. 1A–D) (See Methods). In prior work, we observed that LSS provided a greatly expanded dynamic range of assessing genes at the lowest end of gene expression which we further investigated in the current report^9^. When comparing LSS and gene expression (RSEM), we observed that mean LSS values were positively correlated with mean gene expression across samples, but the relationship was not linear (Supplementary Fig. 1A). The point in the distribution of RSEM values at which LSS starts to vary provided additive information not otherwise discernable based on the RSEM distribution alone. Specifically, genes with high expression (RSEM > 50) almost always showed high LSS (> 0.9), while genes with very low expression (RSEM < 5) had correspondingly low LSS. In contrast, genes with intermediate expression levels (RSEM 5–50) exhibited a wide range of LSS values (0–1), reflecting variable *on*/*off* states. This sigmoidal relationship suggests that LSS captures information beyond abundance alone, distinguishing structured low-level expression from random background noise. For example, we examined the gene *KRT82* in HNSC cohort to show how LSS complements conventional expression measures (Supplementary Fig. 1B–E). Among four representative samples, those with high or moderate RSEM values showed consistently high LSS and structured coverage profiles (*on* state), whereas two low-RSEM samples differed remarkably. One (TCGA-CN-6997) retained high LSS, while the other (TCGA-CN-4733) showed low LSS (random coverage). Despite similarly low expression levels, LSS clearly distinguished *on* and *off* states, illustrating ability of LSS to capture biologically meaningful variation underlying sparse read coverage. Based on this investigation, we hypothesized that this high-dimensional representation using coverage shapes can better distinguish genes expressed at very low level from a background noise distribution, which is often difficult to separate using traditional techniques. Additionally, we showed that LSS is largely insensitive to exon GC content and gene length, indicating that it captures biologically relevant coverage patterns from RNA-seq data while minimizing the influence of these technical biases (Supplementary Fig. 1F–H).

In order to interrogate LSS values consistent with the concept of a gene that is either in the *on* or *off* state, we considered *XIST* gene which has been previously studied in the context of a gene that is expected to be inactive in males, representing a utility gene for investigation of true *on* and *off* status. Base-level RNA-seq coverage of *XIST* was at least ten-fold higher in females (*on* state) than in males, and was zero or near zero in most males, consistent with an *off* state (Fig. 1E and F). Considering the LSS transformation of *XIST* demonstrated a bimodal distribution strongly associated with gender and distinctively divided at a threshold of 0.6 (Fig. 1G). This threshold suggests that samples with LSS > 0.6 are in an *on* state and those with LSS < 0.6 are likely an *off* state. Interestingly, although the expectation is that *XIST* is off in all males, some low number of reads is measurable in approximately 25% of male samples for some genic regions or across an entire gene such that the state of biologically off includes at least some background RNA measurement error (Fig. 1F). However, these background reads present noisy, inconsistent patterns, resulting in very low LSS values, and thus the distinction between male and female patients becomes more discernible by the LSS distribution. Also, we observed a similar gender-associated distribution in genes on the Y chromosomes although the precise optimal cut point varied as a function of absolute expression of the gene. Higher values of RSEM gene expression generally translated into slightly higher cut points (> 0.6) and lower values of RSEM translated to LSS cut points slightly lower (< 0.6). However, on average, 0.6 was a convenient threshold with reasonable performance across the range of genes on the Y chromosome, supporting a decision as to which genes could be effectively distinguished as *on* or *off* (Supplementary Fig. 2A–C). For these Y chromosome genes, although female patients also demonstrated a low background signal, the sequencing read mapping did not reflect the pattern associated with transcript shape (Supplementary Fig. 2D–I). In our previous study, we validated the assumption that RNA-seq samples present a common coverage structure using both technical and biological replicates from human bronchial epithelial (HBe) cells^9^. While the comparison between biological replicates yielded high LSS values, indicating a strongly shared structure among the samples, significantly lower LSS values were observed between random and biological replicates, with a distribution primarily below 0.6. This pattern implied that background noise, which typically results in low coverage, was unlikely to yield an LSS value above 0.6, regardless of the level of expression. This confirmed the selected threshold can be systematically applied as a reliable criterion for LSS to distinguish between *on* and *off* states of gene expression. Based on this threshold, in this study, we defined the offonome as the set of genes showing an *off* status in at least 20% of the cohort, regardless of their expression levels. Conceptually, genes with more than 20% of samples in the *off* status may be biologically interesting, as this level of recurrence suggests a non-random pattern that is unlikely to arise simply from outlying or abnormal samples. We therefore believe that such genes are more likely to reflect meaningful *on*/*off* states. This definition identified 5,851 offonome genes in the HNSC cohort, which will be discussed in more detail in the following sections. We note that 66% of these genes overlap with those that would be filtered by the edgeR package using its default settings (min.count = 10, min.total.count = 15, min.prop = 0.7, and large.n = 10). Taken together, by using LSS, all genes can be defined as being either *off* or *on* which is a classification we define as the offonome. Since LSS is a continuous function between 0 and 1, gene expression may alternatively be described by the LSS without enforcing a dichotomous classification. Within the continuum of expression, the approach above offers an unbiased and robust measure as to the lowest level of gene expression for which LSS can reliably measure expression above background noise, a property with potential utility when considering very low gene expression. We note that the use of LSS does not require transformation to a binary term (*on*/*off*) to retain its utility for describing gene expression at very low levels, but for the purposes of this report we find this transformation useful particularly for visualization.

### Offonome of lung adenocarcinoma (LUAD) and lung squamous cell carcinoma (LUSC)

**Fig. 2:**
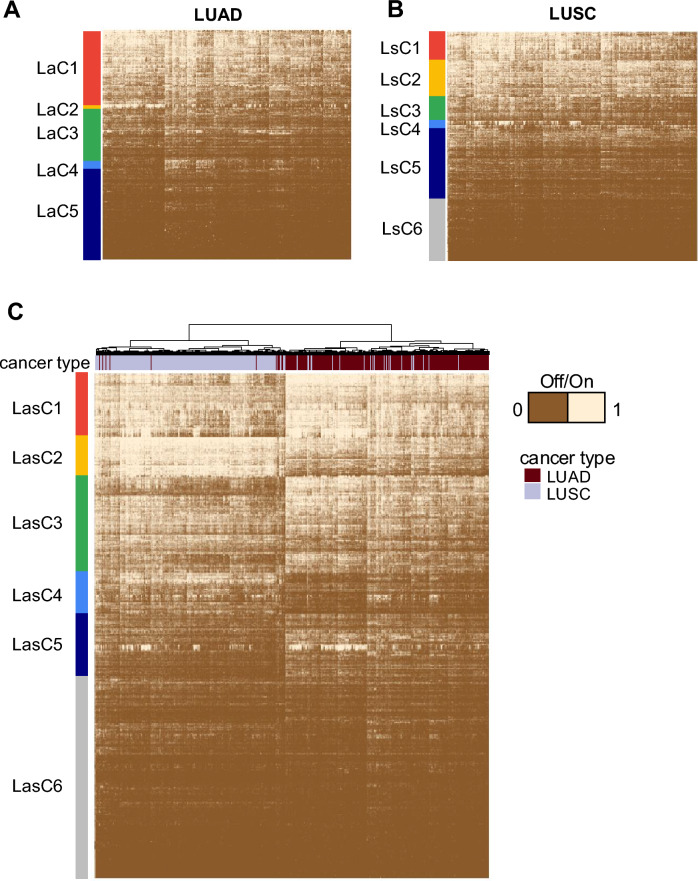
Offonome of Lung adenocarcinoma (LUAD) and Lung squamous cell carcinoma (LUSC). **a** The heatmap showing the result of LSS clustering of offonome for LUAD tumor samples. **b** The heatmap showing the result of LSS clustering of offonome for LUSC tumor samples. **c** The heatmap showing the result of LSS clustering for combined offonome of LUAD and LUSD (5,742 genes). Cancer type is designated by annotation bar: brown for LUAD and blue for LUSC. For all heatmaps, genes with LSS values greater than 0.6 are considered *on* status (1 in heatmap) otherwise a gene is considered *off* status (0 in heatmap). *LaC* lung adenocarcinoma cluster, *LsC* lung squamous cell carcinoma cluster, *LasC* lung adenocarcinoma and lung squamous cell carcinoma combined cluster. Clusters annotation was defined based on the hierarchical cluster tree and manipulated to emphasize small clusters (LaC2 and LsC4) showing distinctive *on* and *off* status across samples.

We applied LSS to two cancer types, LUAD and LUSC, sharing the same anatomic site with different morphologic types. We started with all protein coding genes in the 419 LUAD samples and 415 LUSC samples, assigning each sample/gene as either *on* or *off*. For each tumor type, genes were retained for the offonome if more than 20% of the samples were in *off* state, emphasizing a set of genes that would often have been removed from similar analysis due to low expression (Supplementary Fig. 3A and B). The total offonome genes (N=5,434 for LUAD and N=5,292 for LUSC) includes 4,984 genes (86% of total) that were common to LUAD and LUSC with 308 LUSC-specific genes (5.3%) and 450 LUAD-specific genes (7.8%) (Supplementary Fig. 3C). We employed the hierarchical clustering method to investigate the properties of LSS (including binary transformation to *on*/*off* state) to characterize specific cancer types with a focus on genes meeting our definition of offonome. LUAD and LUSC offonome revealed 5 clusters (LaC1–5) and 6 clusters (LsC1–6) respectively (Fig. 2A and B, Supplementary Fig. 3D and E). Ontologies associated with genes defining each of the clusters revealed both expected and cohort specific biological pathways with high statistical significance (FDR < 1.0E-03, Table 1).

**Table 1:** Gene ontology analysis by cohort and cluster.

Cohort	Cluster name	Ontology	count	p value	fdr
LUAD	Cluster 1 (LaC1)	Developmental process	538	3.01E-12	3.93E-09
		Transmembrane transport	158	2.89E-11	3.48E-08
		Nervous system development	242	2.35E-10	1.84E-07
	Cluster 2 (LaC2)	Cilium movement	10	3.69E-10	5.78E-06
		Microtubule-based movement	13	4.99E-10	3.91E-06
	Cluster 3 (LaC3)	Ion transport	117	2.69E-11	8.44E-08
		Anatomical structure development	349	1.19E-10	2.34E-07
		Trans-synaptic signaling	58	1.43E-10	2.49E-07
		Chemical synaptic transmission	55	5.86E-10	5.74E-07
	Cluster 4 (LaC4)	Regulation of transcription by RNA polymerase II	46	2.12E-07	1.11E-03
		snRNA 3’-end processing	6	5.16E-07	8.98E-04
	Cluster 5 (LaC5)	Detection of chemical stimulus involved in sensory perception	360	5.57E-180	8.74E-176
		G protein-coupled receptor signaling pathway	463	2.53E-145	4.41E-142
		Nervous system process	475	5.61E-128	8.79E-125
		Keratinization	36	5.64E-13	7.30E-11
		Natural killer cell activation involved in immune response	15	1.39E-07	8.59E-06
		Type I interferon signaling pathway	15	2.14E-05	7.85E-04
LUSC	Cluster 1 (LsC1)	Positive regulation of cytosolic calcium ion concentration	21	1.21E-06	1.11E-03
		Plasma membrane bounded cell projection organization	58	1.92E-05	9.72E-03
		Cell projection organization	58	6.14E-05	2.60E-02
	Cluster 2 (LsC2)	Epithelial cell differentiation	59	7.48E-12	3.91E-08
		Keratinization	19	6.89E-10	1.35E-06
		Intermediate filament cytoskeleton organization	17	8.56E-08	6.10E-05
		Ion transport	77	2.13E-07	1.28E-04
	Cluster 3 (LsC3)	Ion transport	61	1.77E-08	9.24E-05
		Chemical synaptic transmission	28	8.41E-07	1.01E-03
		Regulation of ion transmembrane transport	30	4.99E-06	3.13E-03
	Cluster 4 (LsC4)	Cilium movement	15	1.22E-11	1.92E-07
		Microtubule-based movement	18	1.91E-09	1.49E-05
	Cluster 5 (LsC5)	Nervous system process	310	2.34E-37	1.23E-33
		G protein-coupled receptor signaling pathway	266	1.88E-33	4.91E-30
		Detection of stimulus	165	1.27E-24	1.42E-21
	Cluster 6 (LsC6)	Detection of chemical stimulus involved in sensory perception of smell	249	5.45E-228	8.55E-224
		G protein-coupled receptor signaling pathway	280	3.55E-172	6.18E-169
		Natural killer cell activation involved in immune response	9	1.90E-07	1.64E-05
		Type I interferon signaling pathway	9	7.36E-06	4.69E-04
Lung cancer offonome	Cluster 1 (LasC1)	Cilium movement	28	2.25E-12	1.76E-08
		Microtubule bundle formation	23	3.42E-11	1.79E-07
		Axoneme assembly	20	7.19E-11	2.82E-07
	Cluster 2 (LasC2)	Epidermis development	33	9.58E-14	1.50E-09
		Keratinization	16	7.73E-11	4.04E-07
		Skin development	26	1.39E-10	4.37E-07
	Cluster 3 (LasC3)	Regulation of ion transport	77	1.55E-11	8.09E-08
		Neuron differentiation	97	2.32E-10	6.06E-07
	Cluster 4 (LasC4)	Keratinization	29	5.15E-24	8.08E-20
		Epithelial cell differentiation	53	3.81E-17	1.19E-13
		Intermediate filament organization	17	1.94E-12	3.81E-09
	Cluster 5 (LasC5)	Cellular metabolic process	113	3.77E-11	2.96E-07
		Ion transport	72	1.48E-10	1.78E-07
		Inorganic ion transmembrane transport	43	1.23E-09	9.17E-07
	Cluster 6 (LasC6)	Detection of chemical stimulus involved in sensory perception	364	1.13E-191	1.77E-187
		G protein-coupled receptor signaling pathway	479	1.05E-157	2.05E-154
HNSC	Cluster 1 (HNsC1)	Multicellular organismal process	549	1.85E-13	4.14E-10
		Developmental process	482	1.60E-12	2.09E-09
		Ion transport	145	2.24E-12	2.34E-09
	Cluster 2 (HNsC2)	Muscle contraction	38	4.00E-40	3.14E-36
		Muscle cell differentiation	31	2.44E-28	5.47E-25
		Myofibril assembly	20	2.65E-26	5.19E-23
		Actomyosin structure organization	21	3.78E-23	5.38E-20
		Skeletal muscle tissue development	20	1.84E-20	2.22E-17
	Cluster 3 (HNsC3)	Chemical synaptic transmission	41	9.54E-07	3.12E-04
		Ion transmembrane transport	68	2.35E-06	5.94E-04
		Transmembrane transport	86	3.13E-06	7.33E-04
		Nervous system process	98	3.74E-06	8.61E-04
	Cluster 4 (HNsC4)	G protein-coupled receptor signaling pathway	136	3.96E-12	2.48E-09
		Sensory perception	117	6.14E-12	3.56E-09
		Neuropeptide signaling pathway	25	1.07E-07	3.09E-05
	Cluster 5 (HNsC5)	Detection of chemical stimulus involved in sensory perception	340	2.51E-197	3.93E-193
		Positive regulation of peptidyl-serine phosphorylation of STAT protein	12	2.36E-07	1.50E-05
		Natural killer cell activation involved in immune response	12	1.44E-06	8.12E-05
		B cell proliferation	15	1.27E-05	5.50E-04
		Type I interferon signaling pathway	12	9.95E-05	3.43E-03
Integrative offonome	Cluster 1 (IC1)	Cell adhesion	91	1.42E-08	1.01E-05
		Microtubule bundle formation	23	4.12E-07	2.31E-04
		Inorganic ion transmembrane transport	67	7.61E-07	3.73E-04
	Cluster 2 (IC2)	Epidermis development	65	1.08E-24	1.70E-20
		Keratinization	33	2.28E-20	1.19E-16
		Intermediate filament organization	20	1.00E-10	9.81E-08
	Cluster 3 (IC3)	Cilium movement	26	2.03E-09	1.77E-06
		Microtubule-based movement	37	9.35E-08	4.58E-05
		Regulation of ion transport	51	2.33E-06	7.78E-04
	Cluster 4 (IC4)	Nervous system process	198	2.13E-17	3.33E-14
		Transmembrane transport	161	3.15E-13	3.09E-10
		G protein-coupled receptor signaling pathway	156	5.60E-12	3.99E-09
		Chemical synaptic transmission	74	1.00E-11	6.30E-09
		Muscle system process	40	8.51E-05	6.61E-03
		Striated muscle adaptation	10	1.46E-04	1.01E-02
	Cluster 5 (IC5)	Detection of chemical stimulus involved in sensory perception	340	1.80E-202	1.41E-198
		G protein-coupled receptor signaling pathway	422	2.91E-164	5.71E-161
		Regulation of peptidyl-serine phosphorylation of STAT protein	14	1.34E-08	1.03E-06
		Natural killer cell activation involved in immune response	14	2.94E-08	2.15E-06
		G protein-coupled serotonin receptor signaling pathway	15	1.47E-07	9.62E-06
		Type I interferon signaling pathway	14	4.30E-06	2.17E-04

Having investigated the LUAD cluster, we focused on LaC2, comprised of approximately 90 genes primarily related with cilia and microtubules (Fig. 2A and Table1). LaC2 demonstrates genes in the *on* state for roughly 20% of samples and *off* for the remaining 80%. Notably, cilia are generally absent in malignant cells, such that detection of these genes suggests infiltration of non-malignant epithelium^14^. Therefore, LaC2 offered strong evidence for infiltration of a specific cell type of normal epithelium. In contrast to infiltrating normal epithelial cells, LaC5 is broadly defined by immune cell infiltration. Genes within this cluster include those associated with immunostimulatory type I interferon signaling pathway and natural killer cell activation in a pattern known to be associated with lung cancer^15^. Offonome patterns in LaC4 demonstrated genes more likely to be intrinsic to a proliferative malignant phenotype including the RNA polymerase II complex whereas LaC1 implicated gene ontology associated with differential pulmonary epithelial development across the cohort. LUSC independently recaptured a cluster enriched in cilium movement (LsC4) and immune activation (LsC6) (Table 1), similar to what we observed in LUAD^15^. In contrast to LUAD, LUSC clustering demonstrated ontologies associated with squamous epithelium including keratinization (LsC2) and epithelial ion transport and chemical synaptic transmission (LsC3).

Having considered two separate subtypes of non-small cell lung cancer independently, we considered a union set of lung cancer offonome (n = 5,742 genes) in the merged cohort of LUAD and LUSC. Not unexpectedly, unsupervised clustering recaptured morphologic classification of LUAD versus LUSC (Fig. 2C), resulting in two dominant clusters that were specific to each cancer type. One of them contained 402 samples (17 of which were LUAD and 385 of which were LUSC) and the other had 438 samples (30 of which were LUSC and 402 of which were LUAD). Notably, these offonome-based clusters showed 94% of classification rate in assigning morphologic subtypes of lung cancer, suggesting the potential applicability of LSS with genes in the low-end of expression level to cancer type characterization. In addition to distinguishing LUAD from LUSC, union clustering re-emphasized patterns of ontologies of offonome seen when the tumor groups were clustered separately including cilia movement, microtubule bundle formation (LasC1), keratinization (LasC2), ion transport (LasC3/5), and lung differentiation (LasC4) (Table 1). Selected patterns such as keratinization distinguished the major classes of LUAD and LUSC, with nearly all LUSC samples demonstrating relatively higher expression. Interestingly, patterns that distinguished the major groups of LUAD also appeared to vary within the group. For example, the squamous-defining cluster of keratinization genes (LasC2) showed variable expression within LUAD, suggesting a more squamous-like form of LUAD which has been repeatedly reported^16^. Union clustering of LUAD/LUSC identified at least two additional statistically significant ontologies in LasC6 relevant to both histologic variants: detection of chemical stimulus involved in sensory perception and G protein-coupled receptor signaling pathway. In addition, approximately 35% of the LUAD/LUSC offonome genes were characterized as *off* in most samples. In other words, these genes are, to the best of our ability to estimate, not expressed at all.

### Offonome of head and neck squamous cell carcinoma (HNSC)

**Fig. 3:**
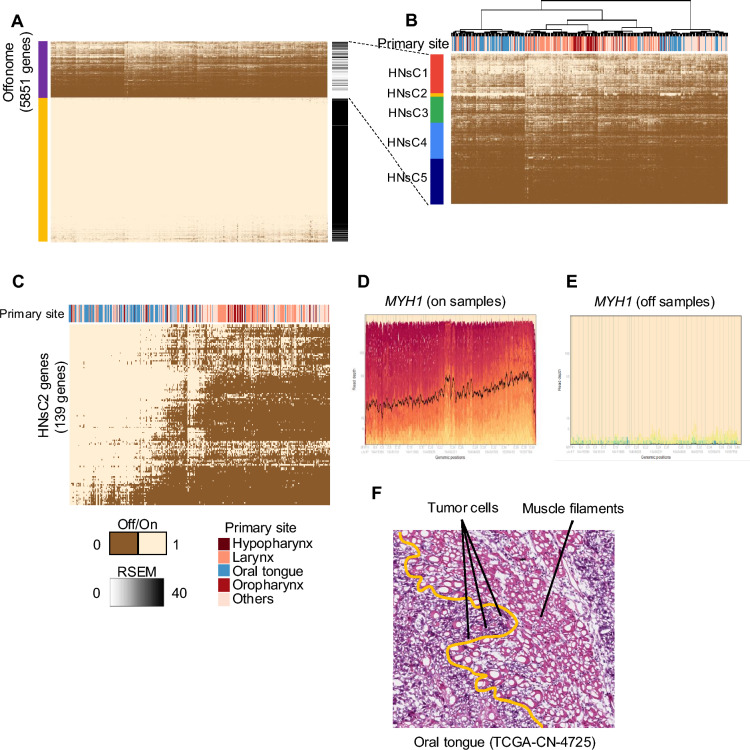
Offonome of Head and Neck Squamous Cell Carcinoma (HNSC). **a** Heatmap of LSS clustering result for all genes (20,511) of HNSC tumor samples. The purple cluster indicates offonome genes and left side annotation indicates averaged RSEM quantification value of each gene. **b** Focused view of HNSC offonome (5,851 genes) group. For all heatmaps, genes with LSS values greater than 0.6 are considered *on* status (1 in heatmap) otherwise a gene is considered *off* status (0 in heatmap). Heatmap column annotation shows the primary site of HNSC tumor sample according to colors and HNsC indicates head and neck squamous cell carcinoma cluster. Clusters were defined based on hierarchical clustering dendrogram. **c** Re-clustering result of the data from HNsC2 gene list and LSS value. Heatmap annotation also shows the primary site of HNSC tumor sample according to colors. **d** Plot shows the single base resolution RNA-seq coverage of the MYH1 gene (included in HNsC2) for subset of samples having on status for MYH1 gene. **e** Plot shows the single base resolution RNA-seq coverage of the MYH1 gene (included in HNsC2) for subset of samples having off status for MYH1 gene. **f** H&E slide data of HNSC tumor samples (TCGA-CN-4725). Based on the orange line, the majority of right-side shows muscle filament and the tumors were infiltrated into muscle filaments.

We considered HNSC to further characterize the properties of genes expressed in the *on*/*off* spectrum in a second anatomic site tumor of the aerodigestive system sharing morphology with LUSC. Using the same filtering strategy applied to lung cancer, we identified 5851 offonome genes with 5 gene clusters (HNsC1–5) (Fig. 3A). Perhaps not unexpectedly we observed sub-clustering of tumors based on anatomic site, with most of the oropharynx tumors clustering together (Supplementary Fig. 4A and B). The finding of oropharynx tumors as a cluster using the offonome mirrors similar work using conventional expression measures due HPV etiology associated with this anatomic subsite (Fig. 3B). Notably, we observed a subset of oral cavity tumors showing a tight cluster in a manner which lacked an obvious a priori etiology but clearly had high expression of a set of genes strongly associated with HNsC2 (Fig. 3B). Investigation of this gene set revealed a plausible biologic basis for the cluster related to muscle development and function (Table 1). We hypothesized that contaminating nonmalignant muscle cells infiltrating tumors of the tongue formed the basis of this cluster. This signature was absent in anatomic sub-sites not as often associated with muscle involvement such as the larynx, hypopharynx, and oropharynx (Fig. 3C). The RNA-seq pileup for HNsC2 demonstrated full-length transcript coverage with a high density of mapped reads, which we referred to as high level gene coverage. This pattern was evident for many genes, including *MYH1*, a gene associated with muscle function, which showed abundant read coverage across its entire transcript in the *on* samples. In contrast, these genes displaed a complete absence of expression in the *off* samples (Fig. 3D and E). We reviewed H&E samples from representative cases and confirmed the obvious presence of muscle filaments in corresponding samples and absence in representative sample not associated with that cluster (Fig. 3F). In addition to a strong signal from muscle cell infiltration, we also observed more frequent use of HNsC1 and HNsC3 involved genes associated multicellular organismal process and ion transport in HNSC from larynx and tonsil than other primary sites. Unlike LUSC, HNSC samples provided no evidence for a cluster associated with abundance of cilia since cilia are largely absent in these tissues. The other clusters, HNsC4 and HNsC5, present similar ontologies as with lung cancer, mostly associated with inflammatory infiltrates including type I interferon signaling pathway, B cell proliferation and natural killer cell activation.

### Integrative offonome for three tumor types

**Fig. 4:**
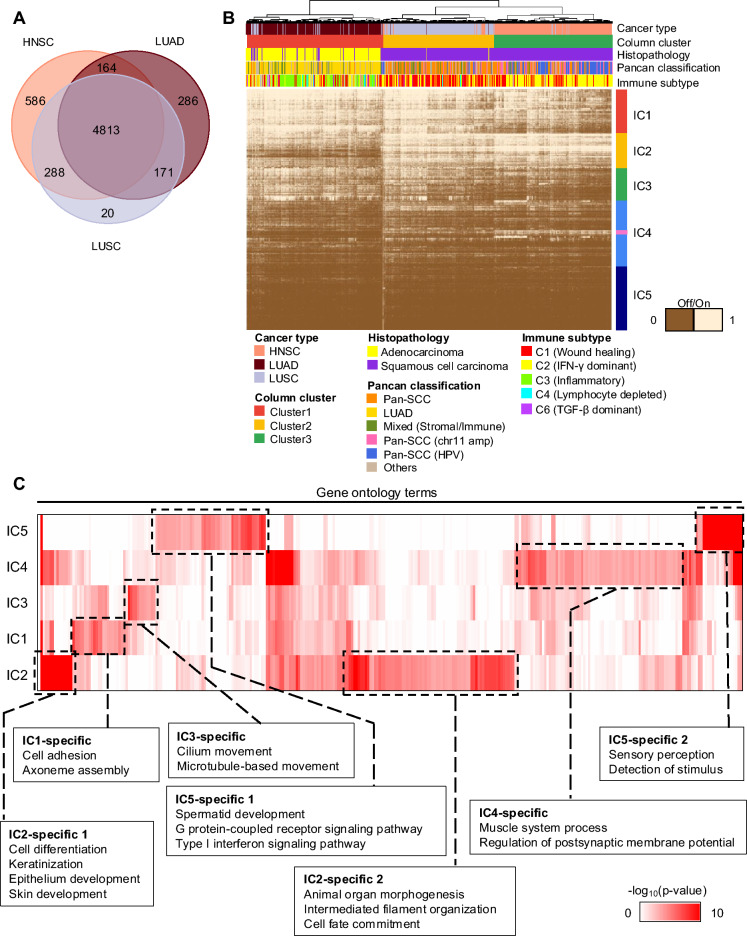
Integrated analysis for 3 tumor types using Offonome. **a** Venn diagram comparing the number of offonome genes across HNSC, LUAD and LUSC (total 6,328 genes). **b** The result of LSS clustering for integrated offonome (6,328 genes). With the value of LSS, *on* status (1 in heatmap) is granted to genes if they have over 0.6 LSS value or otherwise, *off* status (0 in heatmap) is granted to genes. Heatmap column annotation from the top shows TCGA cancer type, column cluster, histopathology information, Pancancer classification and immune subtype information. Heatmap row annotation indicates clusters of genes and pink color in IC4 indicates HNSC-specific keratinization-related gene cluster. **c** Comprehensive analysis of significantly enriched gene ontology terms in integrated tumor clusters. The red color indicates the value of -log_10_(p-value) from each gene ontology terms and columns indicate individual gene ontology terms.

Having considered genes within two anatomic sites and across two morphologic subtypes, we extended the analysis to consider both anatomic site and morphology in the same experiment. We constructed an integrative offonome by taking the union of offonome genes from LUAD, LUSC, and HNSC (n = 6,328 genes) (Fig. 4A). Among these, 4813 genes (76%) were shared across three tumor types, with many exhibiting little or no evidence of expression in any of the samples. For the pairwise comparisons, 288 genes (6.1%) were common to both LUSC and HNSC, while a significantly smaller fraction, 171 genes (2.7%), was shared between the LUAD and LUSC, and 164 genes (2.6%) were found in both LUAD and HNSC. Focusing on genes uniquely associated with a single anatomic-histologic category, 586 genes (9.2%) were specific to HNSC, 286 genes (4.5%) were exclusive to LUAD, and LUSC had the smallest unique set with only 20 genes (0.3%).

We identified 5 gene clusters (IC1-5) based on an unsupervised clustering of the union of the three tumor’s offonome (Fig. 4B). Interestingly, the most prominent clusters separated by the uppermost branches of the unsupervised dendrogram were associated with their native anatomic and morphologic classifications: squamous cancer versus adenocarcinoma (IC2), and lung cancer versus head and neck cancer (IC1). Previous pancancer studies have investigated cancer subtype classification based on a similar or even advanced clustering approach using gene sets filtered in more conventional manners^17,18^. While direct comparison was difficult due to variations in clustering techniques and the number of tumor types considered between the pancancer studies and our own analysis, we highlighted that our offonome-based clustering, which utilized LSS value of genes in a low range of read coverage, effectively distinguished different tumor types. We observed that the offonome domain appeared to be particularly useful for separating HNSC and LUSC (Fig. 4B). To see if there were distinctive patterns of clustering with two gene sets (offonome vs. conventional gene set excluding low-expressed genes), we applied the same clustering strategy with the same set of samples with the only difference being the genes included. For the conventional gene set, we included the 15,363 genes used from the previous TCGA pancancer analysis^17^. The case where genes overlapped the most between the offonome and TCGA was when using the integrated offonome; nevertheless, more than 75% of offonome genes were specific to our study (Supplementary Fig. 5A–E). When we investigated the RSEM of genes specifically found in each offonome (Specific), they generally displayed much lower expression profiles than genes overlapped with TCGA pancancer gene set (Common) (Supplementary Fig. 5F–H). The data showed that our offonome gene definition captured many genes that were previously excluded from the pancancer project due to mean expression close to zero, although some outlier genes could reach RSEM values as high as ~10^6^. Additionally, the current offonome definition also included genes that were not excluded from the pancancer projects (HNSC offonome: 1,367 genes, LUAD offonome: 891 genes, LUSC offonome: 854 genes) with higher mean expression, but still exhibit low mean expression (RSEM< 10). To further explore the properties of LSS in distinguishing different anatomic and morphology simultaneously, we performed offonome-based hierarchical clustering. As a result, we observed that three tumor types could be classified with the LSS value even though integrative offonome genes were in the low range of expression level. The classification of three tumor types was also observed when we used highly expressed gene set from pancancer study (Supplementary Fig. 5I). This strongly suggests that the low-end spectrum of the expression profile presents opportunities for identification of biologically meaningful information which has been ignored in many studies mainly due to concerns over signal to noise ratio at the low end of gene expression.

In addition to recapitulation of morphology and anatomic site, complementary and robust gene set clusters appeared in the integrated cohort. The biologic foundation of these gene signatures was investigated by clustering GO terms associated with genes from the clusters (Fig. 4C). IC1-specific cluster included cell adhesion and axoneme assembly GO terms which represented cilia production found in lung but not HNSC. Accordingly, IC1 was more prominent in the two lung cohorts and less so in the HNSC samples. Similar to the pattern seen when tumor types were analyzed separately above, we observed enrichment of gene ontologies associated with keratinization and epithelium development in the IC2-specific cluster representing squamous cell carcinoma tumor types. Complimenting IC1, the lC3 cluster clearly captured specific cilia programs noted in the lung cohorts when examined separately and now shown in the combined analysis. The small gene cluster lC4 included the strong muscle signature detected in the HNSC anatomic cohort and confirmed that this signature was absent in lung samples. The lC5-specific cluster, although largely characterized by genes that were entirely *off* in most samples, was defined by statistical enrichment of type I interferon signaling pathway involved in immune response (FDR = 2.17E-04). In summary, unsupervised gene expression of offonome genes appears to capture both expected biologic variation, such as robust classification of known anatomic and morphologic cancer classes, as well as potentially previously unrecognized subgroups.

Examination of member genes of the selected ontologies, such as lC2’s keratinization ontology (FDR = 1.06E-20) revealed canonical targets both for LUSC and HNSC (Supplementary Table 3). Interestingly, although cytokeratins found in IC2 appeared to be shared by both LUSC and HNSC, there was a separate cluster of cytokeratins in IC4 that appeared to distinguish LUSC from HNSC (Supplementary Table 3). Importantly, about 50% of genes in IC4 were in the *off* state in most samples, representing a gene set that would be challenging to capture by other methods. Examples included *KRT82,* which exhibits extremely low read coverage for column cluster 1 (LUAD-enriched) almost approaching zero mean read coverage (Supplementary Fig. 6A). In contrast, samples in cluster2 (LUSC-enriched) and cluster3 (HNSC-enriched) showed higher mean read coverage than cluster1, indicating that both cluster samples likely have an *on* state for the *KRT82* gene (Supplementary Fig. 6B–C). However, in the aspect of *on*/*off* status, a clear *on* status was evident primarily in sample cluster 3 suggesting the ability of LSS separating real signal and noise signal.

We have stated that the offonome represents genes that are frequently excluded from analysis in many prior works. To investigate if another measure of gene expression has similar ability with LSS for assessment of genes expressed at the lowest end of the expression spectrum, we executed unsupervised clustering for the integrated offonome gene set using quantitative estimates of gene expression. Replacing LSS, which is formulated to assess differential gene expression in a manner that might have favorable properties at the lowest levels of gene expression, by the widely used RSEM, log_2_RSEM, TPM, variance stabilizing transformation (VST) and centered log-ratio (CLR) demonstrated a lower clustering accuracy than LSS in identifying both anatomic subtype and morphologic subtype (Supplementary Fig. 7A–E). This strongly suggests that LSS may be preferred for interrogating gene expression near the limit of RNA expression detection such as we have executed in the current study.

## Discussion

In prior work, our group defined a metric, the level of shape similarity (LSS) which characterizes the extent to which the coverage of a gene by RNA-seq is reproducibly captured across samples. We have previously used this metric to filter out genes expressed at very low levels, as such genes can introduce noise and reduce the reliability of downstream analyses^9^. In this study, we consider that LSS might allow an analysis with only genes expressed at low levels that have been removed from consideration, by using their coverage properties as a proxy for quantitative gene expression. Moreover, our approach extends to the binary classification of genes as either “*on*” (expressed) or “*off*” (not expressed) within a sample, which we refer to as the *offonome*. We demonstrate that conventional measures of gene expression based on read counts perform less favorably in the set of genes we selected based on very low levels of gene expression, while LSS provides complementary information of gene status in single sample in cases where expression approaches unreliable levels.

By applying widely used clustering algorithms to the identified offonome, we demonstrated that feature selection steps in gene expression analysis could be reconsidered to allow for more genes to be used at the lower end of gene expression. Using the offonome definition and LSS approach, we identified features that recapitulated classification results previously reported with gene sets that excluded offonome gene properties. The classification methods themselves were left unchanged to enable a direct comparison. In short, our findings show that genes typically filtered out as lowly expressed can contribute meaningfully to classification tasks.

We showed examples in the space of low gene expression of outstanding discrimination between tumors of known anatomic site and morphology. Our analysis revealed that assessment of very low levels of gene expression can elucidate populations of infiltrating normal epithelium (ciliated cells in lung cancers), stromal cells (myocytes in tongue cancers), and inflammatory cells in all tumor types. We highlight the ability to detect very rare cell populations such as natural killer cells. Moreover, we show that integrative offonome-based clustering can characterize genes associated with similar morphologies but different anatomic sites, such as cytokeratins that differ between LUSC and HNSC. The ability to dissect cell types within bulk tumors has previously been reported and generated considerable interest, such as CIBERSORT^19^. While detecting specific cell types is not the primary function of the offonome, the current analysis opens the possibility for a much broader palette of genes with which to execute such deconvolutions.

Accurate tumor classification by unsupervised analysis demonstrates that RNA-seq data contain sufficient information to distinguish tumor types. However, there is an alternative hypothesis that batch effects due to the timing of assays from different tumor types might explain some of the distinguishing ability of unsupervised analysis. Batch effects at the very low end of gene expression such as the offonome spectrum might be of particular concern, noting that assays for the LUAD, LUSC, and HNSC cohorts were produced at slightly different times. Even subtle production batch differences might manifest as differences in low gene expression. Batch concerns are minimized when expression signatures have clear biological interpretation, as in the current experiments. Almost without exception, gene programs detected as differentially expressed in the offonome clusters have previously been reported in the tumor types considered in this analysis such as keratinization, cilia movement, and inflammation^20–25^.

As reported previously, sequencing to 100–150 million filtered reads can present reliable gene detection^26^. However, there’s no one-size-fits-all ground truth for reliable gene detection in bulk RNA-seq. Instead, truth is linked to spike-in-based limits of detection, consortium standards (GTEx) and library-size filtering^27–30^. Otherwise, by leveraging across transcript coverage shape, LSS reduces dependence on fixed count/TPM cutoffs and complex limit of detection rules. Also, it enables LSS as a potential strategy for identifying reliably expressed genes. Conventional filtering procedures in RNA-seq analysis rely on mean expression thresholds and often remove low-abundance genes indiscriminately. This approach may exclude genes that are weakly expressed yet biologically relevant. In contrast, LSS considers the shape of the coverage profile at the base level, allowing it to distinguish between true low-level expression and random background noise. A gene with low average expression can still display a structured coverage pattern in some samples, indicating an *on* state. This property enables the identification of biologically meaningful *on*/*off* dynamics among genes that would typically be discarded by conventional filtering methods. While we are enthusiastic to report the current techniques using LSS, we anticipate that future development will allow robust gene expression quantification at even lower input levels. As currently implemented, the LSS framework works well in bulk RNA-seq data where most of transcripts are covered by read greater than 1× read. As currently implemented, the LSS framework works best when a transcript is sequenced across its full length with a depth greater than 1×. Future approaches could leverage sequencing platforms that fail to cover the entire transcript or that have coverage of < 1 × across the full transcript such as single cell sequencing. In summary, we present a novel framework for the reliable characterization of genes down to levels much lower than have previously been possible using conventional techniques, leading to a set of genes we define as the offonome. The offonome represents a valuable resource for advancing our understanding of tumor biology, offering new insights into the intricate landscape of gene expression at the lowest detectable levels.

## Methods

### Data sources and pre-processing

Previously aligned RNA-seq BAM files were acquired from the Cancer Genome Atlas (TCGA): HNSC (head and neck cancer, n = 514), LUSC (lung squamous cell carcinoma, n = 486), and LUAD (lung adenocarcinoma, n = 509) for a total of N = 1,509 subjects in the study. The sequence reads were aligned against the GRCh37 (hg19) using MapSplice methods as provided by the legacy TCGA public repository^31^. Using the publicly available SCISSOR R package, for each gene, we generated an independent data matrix composed of read counts of each base position in each sample across the length of the gene. This can be considered the per-base-resolution RNA-seq data in a concept similar to the pileup file format^9^. A gene coverage matrix was generated using ‘read_BAM’ function which takes a set of BAM files as inputs and provides an output of a base-level counts matrix whose columns represent samples and rows contain read counts per genomic position of each gene locus. Gene models were obtained based on TCGA hg19 GAF (gene annotation file) limiting the analysis to 20,511 protein-coding genes. Also, we obtained processed RNA-seq which is RSEM data of HNSC, LUAD and LUSC from TCGA Firebrowse (https://gdac.broadinstitute.org/).

### Filtering out degraded samples

Samples with significant RNA degradation were excluded from the analysis using a similar strategy described in the prior SCISSOR report^9^. For each cancer type, we obtained the decay rates for every sample at individual genes, with higher decay values indicating more severe degradation. Next, we identified a set of samples severely degraded genome-widely as follow: (1) For each cancer type, we pooled the decay rates from all samples across all protein-coding genes and sorted them in descending order. (2) Based on previously reported convention, we then identify the value corresponding to the 95th percentile (top 5%) (e.g., the 527,159th ranked value in HNSC). This value serves as the global threshold separating “non-degraded” from “degraded” decay rates^9^. (3) Samples that included more than 10% of degraded genes were considered significantly degraded samples and excluded from further analysis.

### Level of shape similarity (LSS) and identifying offonome

In prior work, we described a gene expression measure based on single base resolution data object called “the level of shape similarity (LSS)” which was originally developed for a filtering strategy^9^. Briefly, aligned reads were selected to generate a per-base object similar in concept to the pileup file format for each gene in each sample. After log10 transformation of each pileup, we computed a k-adjusted mean profile (default k = 1) across samples, requiring at least 5% of samples to show expression. This process prevents unexpressed or noise-dominated genes from being misclassified as *on*. For each gene, pileup data of all samples and mean pileup data were transformed into a high dimensional vector representation with the number of dimensions equal to the base length of the transcript. Each sample was then compared to the mean vector to estimate the cosine similarity. Larger angles from the mean vector indicate that the corresponding sample presents a higher dissimilarity from the other samples, with the primary explanation being absent or very low expression. To present similarity as values, we calculated cosine similarity [cos(angle)] according to angle between the sample vector and the mean vector, which can provide the value of 1 if two vectors are exactly same or the value of 0 if two vectors are totally different. For the genes that are completely unexpressed in all samples, i.e. genes with zero read, they were given the value of 0 as the shape cannot be defined.

### Selection of LSS threshold for the offonome

To determine the gene status of ‘expressed’ (*on*) versus ‘unexpressed’ (*off*) using LSS, we assessed LSS based on known biologic variations. First, we analyzed genes on the sex chromosomes, as many are known to be selectively expressed in either females or males. We collected LSS values of X chromosome and Y chromosome genes from HNSC samples and generated the plot illustrating the distribution of LSS values of each gene with gender information. Then we explored the distribution patterns of all genes from X and Y chromosomes and identified gene sets exhibiting a bimodal distribution. Subsequently, we compared these selected genes with previous results that investigated the range of LSS obtained from randomized gene coverage structures^9^. In our study, we set the criteria LSS 0.6 to distinguish genes’ *on* and *off* status.

### Offonome cluster analysis

In this study, offonome was defined if more than 20% of samples in a given cohort are identified as *off* states and the minimum percentage of samples can be adjusted by individual researchers. We also compared the HNSC offonome as an example with the filtered genes defined by widely used RNA-seq preprocessing methods which are DESeq2 (default option: ≥ 10 reads across all samples) and edgeR (default option: min.count = 10, min.total.count = 15, min.prop = 0.7 and large.n = 10). Using the defined offonome, hierarchical clustering with the LSS values was performed using the Bioconductor package ComplexHeatmap with Ward’s minimum variance method. The identified gene clusters were further assessed by gene ontology (GO) enrichment analysis with PANTHER 17.0 (FDR < 0.05)^32^. Significantly enriched GO terms were analyzed using the R package, Goseq (v1.54.0). For illustration purposes, the gene ontologies associated with biological process were only used with p-values calculated using Wallenius test. Significantly enriched ontologies for at least one tumor type were selected for integrated analysis. Based on the collection of the selected gene ontology list, a matrix composed of – log_10_(p-values) with gene ontologies in columns and integrative clusters in rows was constructed for unsupervised hierarchical clustering.

## Supplementary Information

Supplementary Information.

## Data Availability

All data generated or analyzed during this study are included in supplementary information files.
