# Supplementary material for: The offonome reveals on and off states of gene expression near the detection limit of RNA-seq
Source: Sci Rep. 2025 Nov 28;15:43141. doi: 10.1038/s41598-025-27185-5 (PMC12678786; doi:10.1038/s41598-025-27185-5)
Supplement: Supplementary file 1 — Supplementary Information. [file 41598_2025_27185_MOESM1_ESM.pdf]

Supplementary Table 1. The number of data used in HNSC, LUAD and LUSC cohorts

Cancer type	Total	Degraded sample	Analyzed sample	Degraded sample percentage
HNSC	514	96	418	18.7
LUAD	509	90	419	17.7
LUSC	486	71	415	14.6

Supplementary Table 2. Clinical summary of data used in HNSC, LUAD and LUSC cohorts

	Category	HNSC (418)	LUAD (419)	LUSC (415)
Gender	Male	305	198	311
	Female	113	221	104
Ethnicity	Hispanic or Latino	17	6	7
	Not Hispanic or Latino	372	306	254
	Not reported	29	107	154
Race	American Indian or Alaska native	1	1	0
	Asian	10	7	7
	Black or African American	39	37	20
	White	355	312	281
	Not reported	13	62	107
Clinical stage	Stage I	17	4	2
	Stage IA	0	103	70
	Stage IB	0	111	127
	Stage II	82	1	2
	Stage IIA	0	41	52
	Stage IIB	0	61	82
	Stage III	86	0	2
	Stage IIIA	0	59	51
	Stage IIIB	0	7	15
	Stage IV	0	26	7
	Stage IVA	209	0	0
	Stage IVB	10	0	0
	Stage IVC	4	0	0
	Not reported	10	6	4

Supplementary Table 3. Different set of keratinization related genes in different integrative clusters

Cluster	Keratinization related genes
IC2	*HRNR*, *SPRR2E*, *KRT4*, *SPRR2F*, *LCE1C*, *SPRR2D*, *KRT6B*, *CASP14*, *IL1A*, *KRT74*, *KRT3*, *IVL*, *SPRR2G*, *KRT76*, *ABCA12*, *LCE3E*, *SPRR1A*, *KRT1*, *KLK5*, *SPRR4*, *TGM3*, *LCE1F*, *KRT75*, *LIPK*, *KRT78*, *SPRR3*, *SPRR1B*, *LCE3A*, *SPRR2B*, *KRT6C*
IC4	*KRT84*, *KRT82*, *KRT73*, *LCE3C*, *LCE5A*, *LCE1B*, *LOR*, *LIPN*, *LCE2B*, *LCE2A*, *LCE1E*, *LCE6A*, *LCE2D*, *KRT2*, *LCE1A*, *KRT71*, *KRT72*, *KRT77*, *LCE2C*, *LCE1D*

Supplementary Figure 1 . The characteristic of level of shape similarity (LSS)

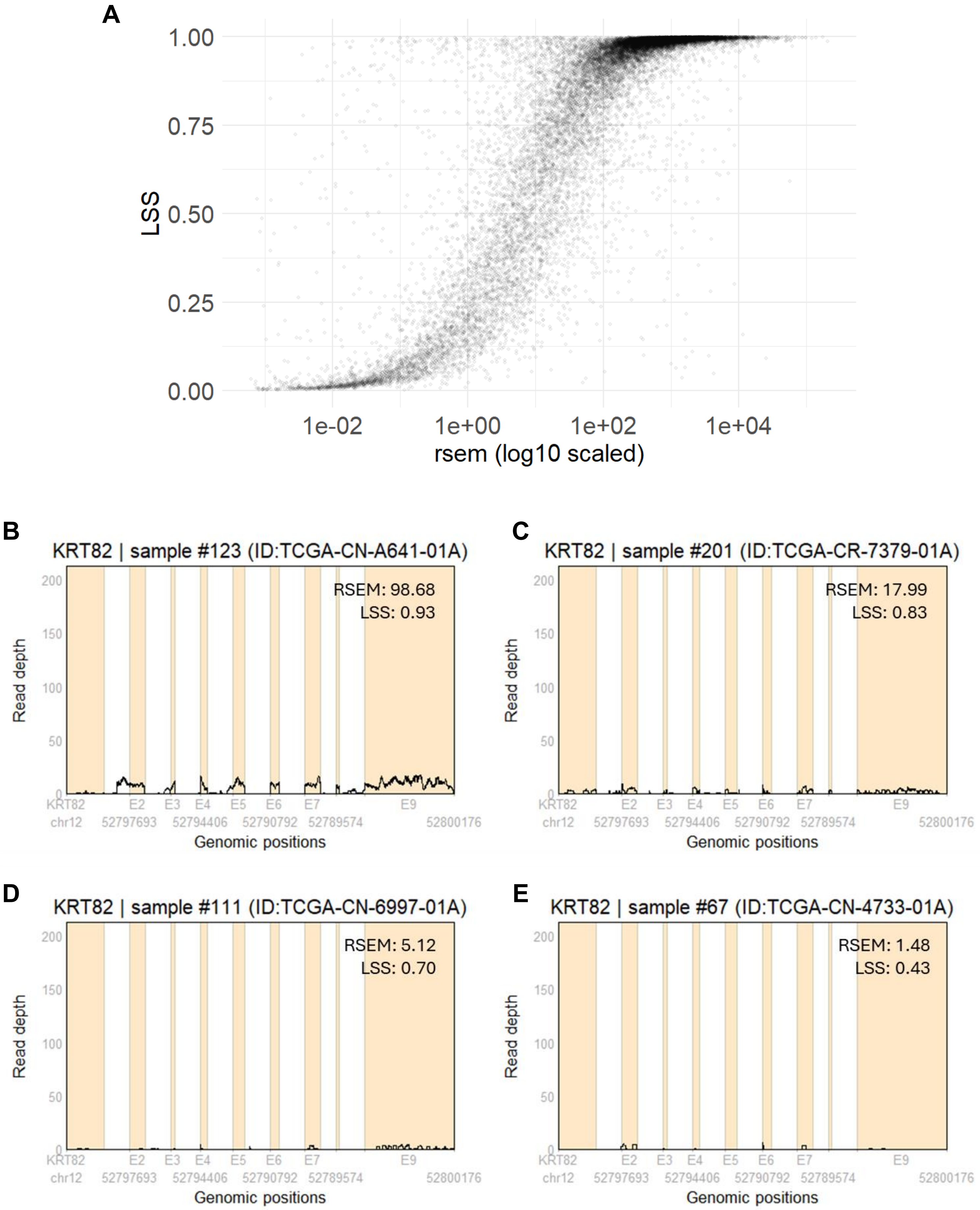

Supplementary Figure 1 . The characteristic of level of shape similarity (LSS)

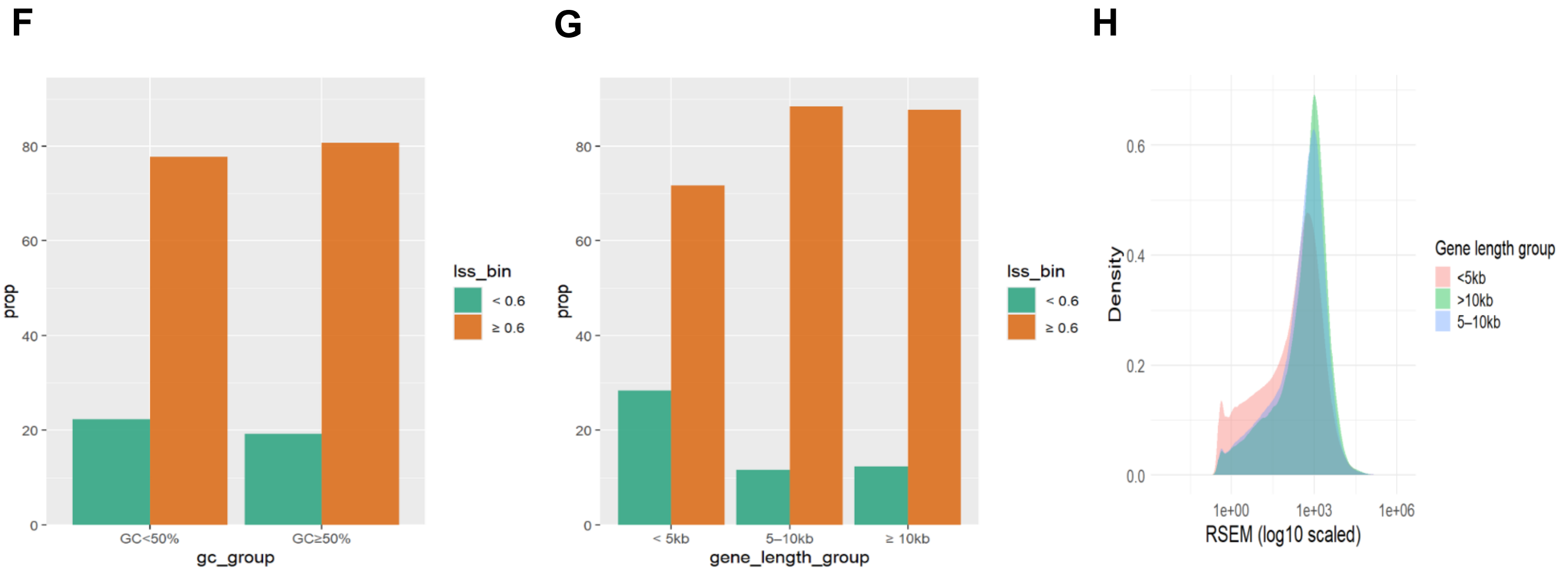

Supplementary Figure 1. The characteristic of level of shape similarity (LSS) (A) Scatter plot for comparing log scaled RSEM and LSS in HNSC data. Each dot indicates gene averaged LSS and RSEM for filtered HNSC samples (418). (B-E) RNA-seq read coverage profiling of TCGA HNSC data for *KRT82* gene. X-axis indicates single base genomic region of *KRT82* including exon (light orange box) and intron (white box). Y-axis read coverage of each single nucleotide. RSEM normalized counts and LSS information were added upper right region of each plot. (F-H) Percentage of on/off genes by exon GC content and gene length. The proportion of genes classified as on (LSS > 0.6) or off was calculated within groups defined by exon GC content (F) and gene length (G). Genes were stratified into two GC content categories (GC > 50% and GC ≤ 50%) and three gene length categories (< 5 kb, 5–10 kb, and > 10 kb). (H) Distribution of RSEM according to gene length.

Supplementary Figure 2 . Application of LSS for Y chromosome genes and simulated genes

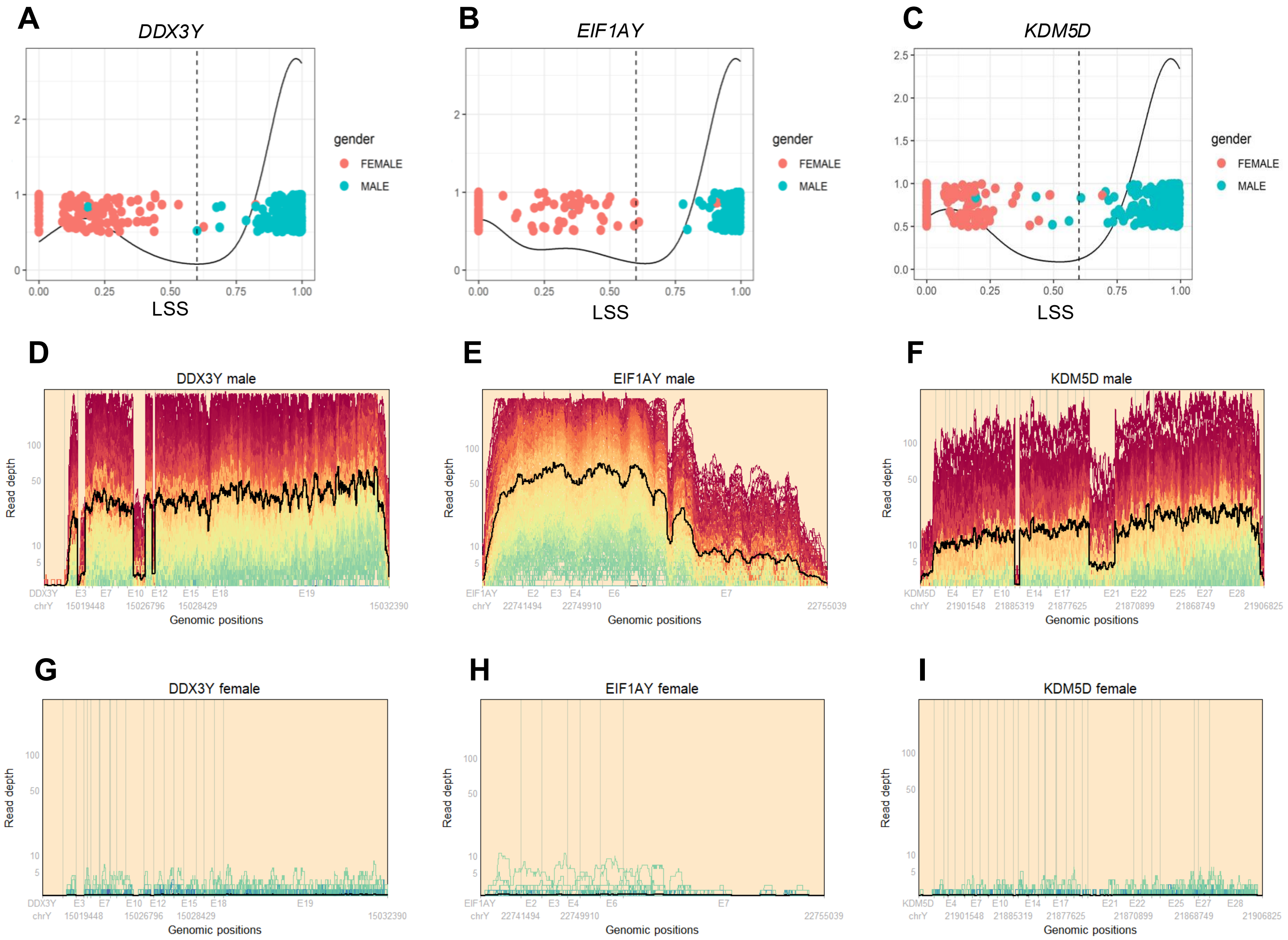

Supplementary Figure 2 . Application of LSS for Y chromosome genes and simulated genes. The distribution of LSS of Y chromosome located genes which are (A) *DDX3Y*, (B) *EIF1AY* and (C) *KDM5D* gene. Vermilion dots indicate male samples, and sky-blue dots indicate female samples. Dash line shows 0.6 LSS value which is set point of distinguishing genes' *on* and *off* status in this study. To avoid the overlap, we intentionally separated each sample dot by adding random scatter in the Y-axis (D-F) We constructed single base resolution RNA-seq pileup data to directly compare the read depth and shape of transcript of male and female for *DDX3Y*, *EIF1AY* and *KDM5D* genes. X-axis indicates genomic position of each gene and Y-axis indicates read depth of each genomic position.

Supplementary Figure 3. LSS clustering for all genes of LUAD and LUSC

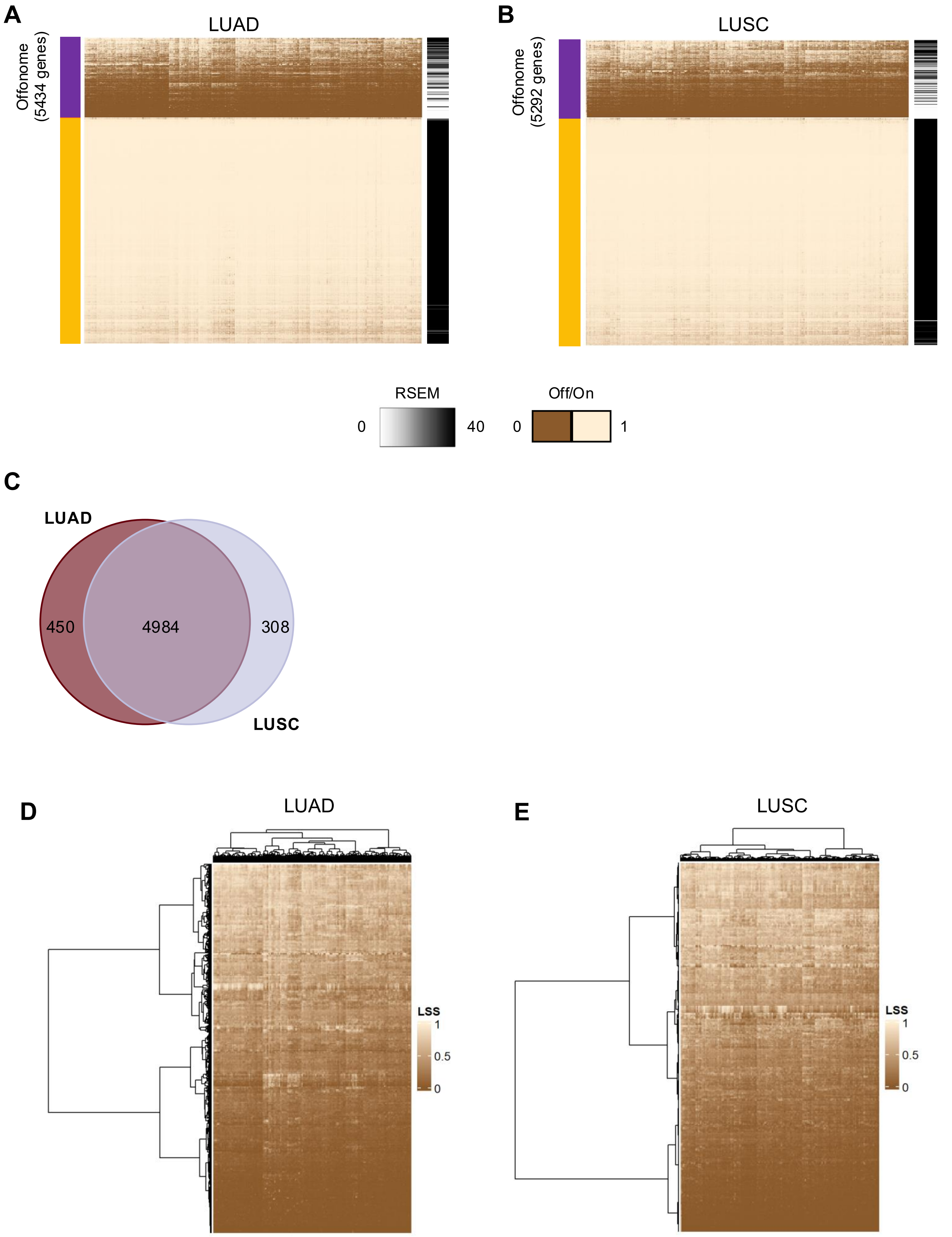

Supplementary Figure 3. LSS clustering for all genes of LUAD and LUSC (A,B) We show the heatmap of LSS clustering result for protein coding 20511 genes of LUAD and LUSC. To enhance the interpretation of the *on* and *off* status in this heatmap, we designate LSS as 1 when LSS is above 0.6, or as 0 when LSS is below 0.6. By indicating the purple cluster of left side of heatmap, we identified offonome of LUAD (5434 genes) and LUSC (5292 genes). Row annotation of right side indicates averaged RSEM value of each gene. (C) We compare the number of offonome of LUAD and LUSC by showing the Venn diagram. (D, E) Focused view of LUAD and LUSC offonome clustering results with row and column dendrogram.

Supplementary Figure 4. LSS clustering for HNSC

A

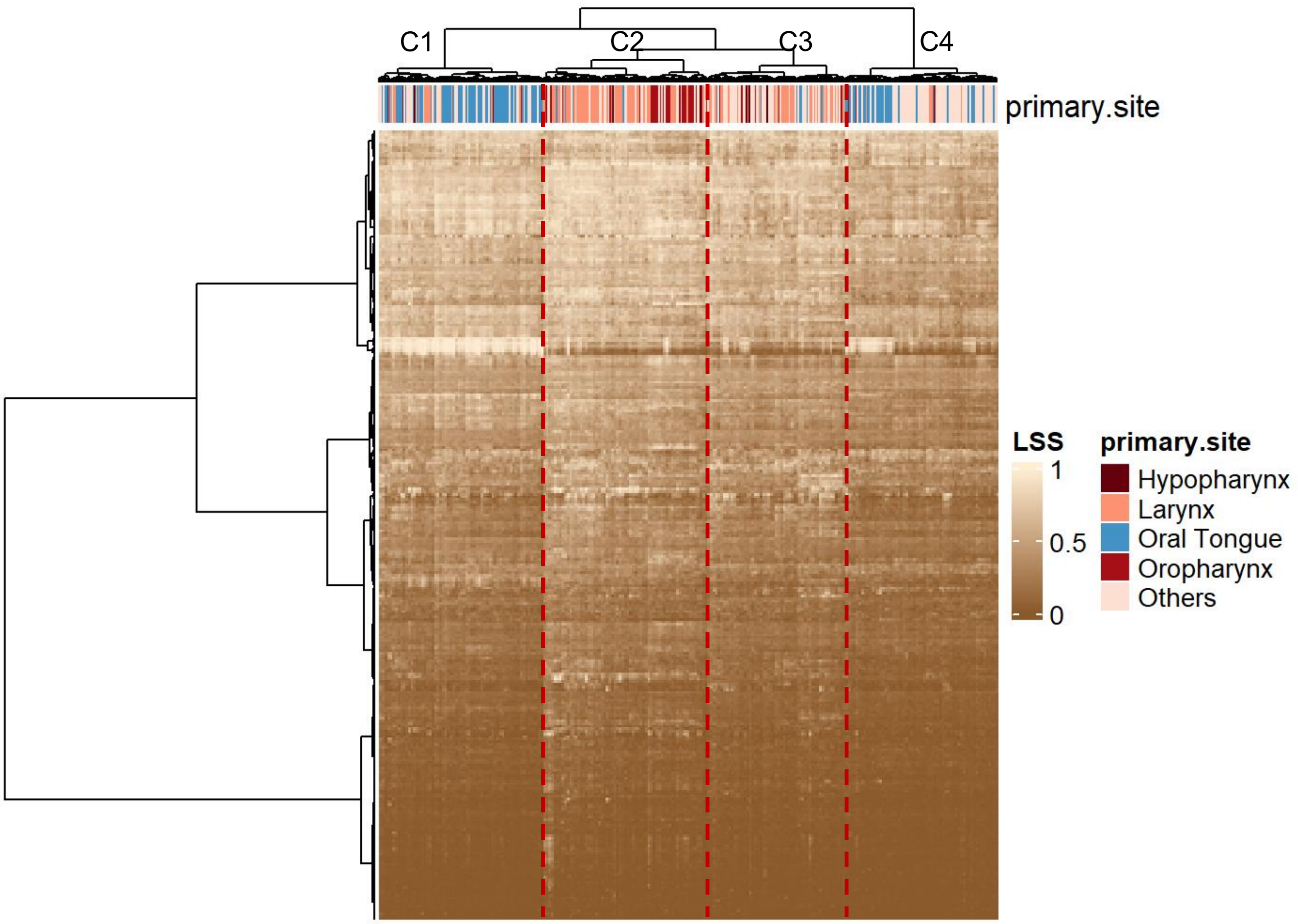

B

	C1	C2	C3	C4
**Hypopharynx**	2	2	3	0
**Larynx**	13	51	32	4
**Oral Tongue**	60	4	7	33
**Oropharynx**	3	26	5	3
**Others**	33	28	46	63

Chi-square test (p-value < 2.2e-16)

Supplementary Figure 4. LSS clustering for HNSC (A) We show the heatmap of LSS clustering result for HNSC offonome with column and row dendrogram. In this figure, we designate LSS as 1 when LSS is above 0.6, or as 0 when LSS is below 0.6. We also indicate the column cluster as C1, C2, C3 and C4. (B) The association of primary sites of HNSC samples in column cluster indicated in (A). Based on this table, we executed chi-square test to see if there is a significant relationship between column cluster and primary site.

Supplementary Figure 5. Clustering analysis using genes selected with conventional filtering criteria

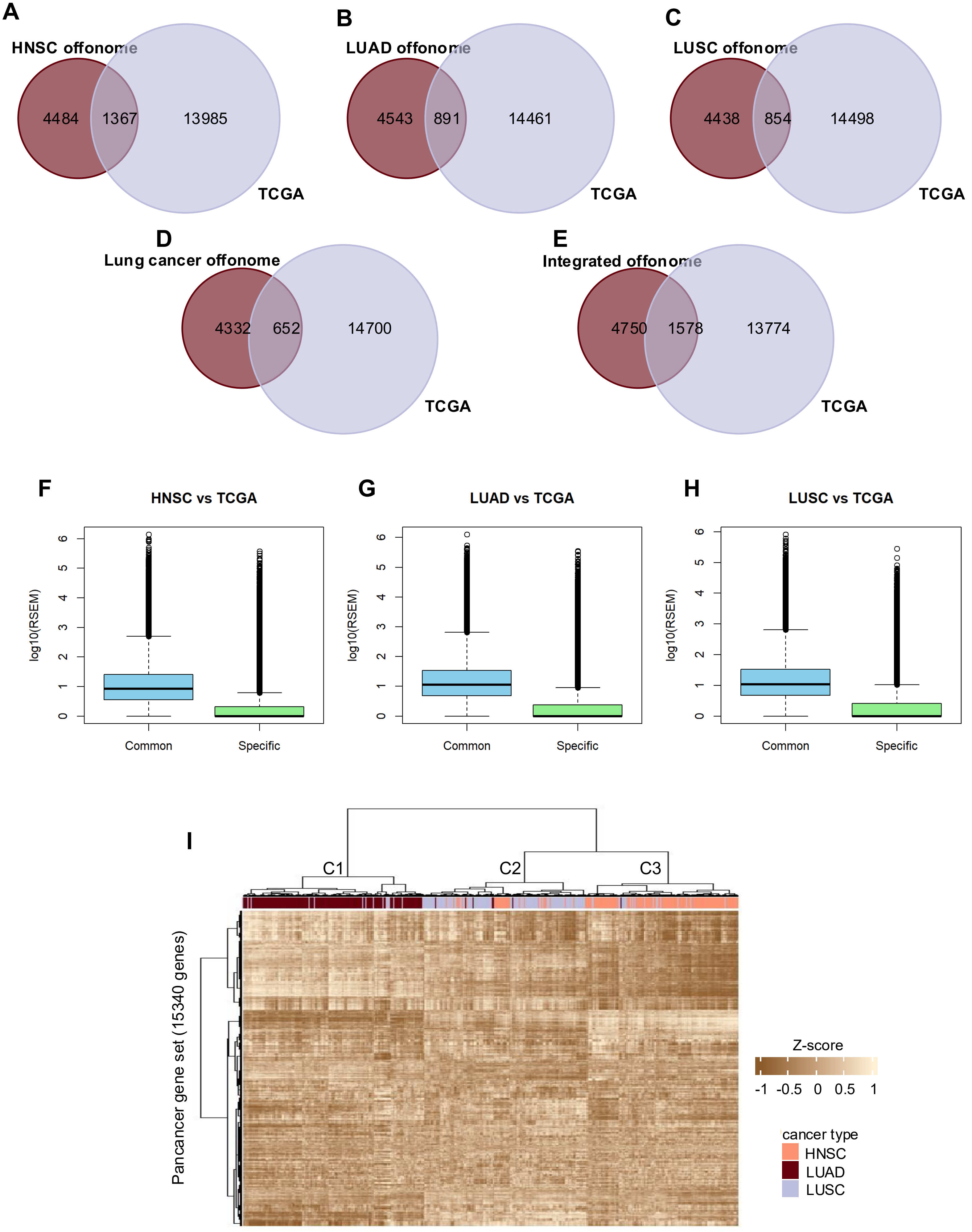

Supplementary Figure 5. Clustering analysis using genes selected with conventional filtering criteria (A-E) Venn diagrams comparing the number of genes of different offonome (A:HNSC offonome, B:LUAD offonome, C:LUSC offonome, D:LUAD and LUSC integrated offonome, and E:HNSC, LUAD and LUSC integrated offonome) and gene set from TCGA pan-cancer analysis study. (F) The heatmap illustrates the outcomes of unsupervised clustering, displaying row-scaled log_2RSEM values for 15,340 TCGA pan-cancer genes across 1,252 samples. These samples include 418 from Head and Neck Squamous Cell Carcinoma (HNSC), 419 from Lung Adenocarcinoma (LUAD), and 415 from Lung Squamous Cell Carcinoma (LUSC).

Supplementary Figure 6. Lowly expressed keratinization genes associated with cancer type clustering

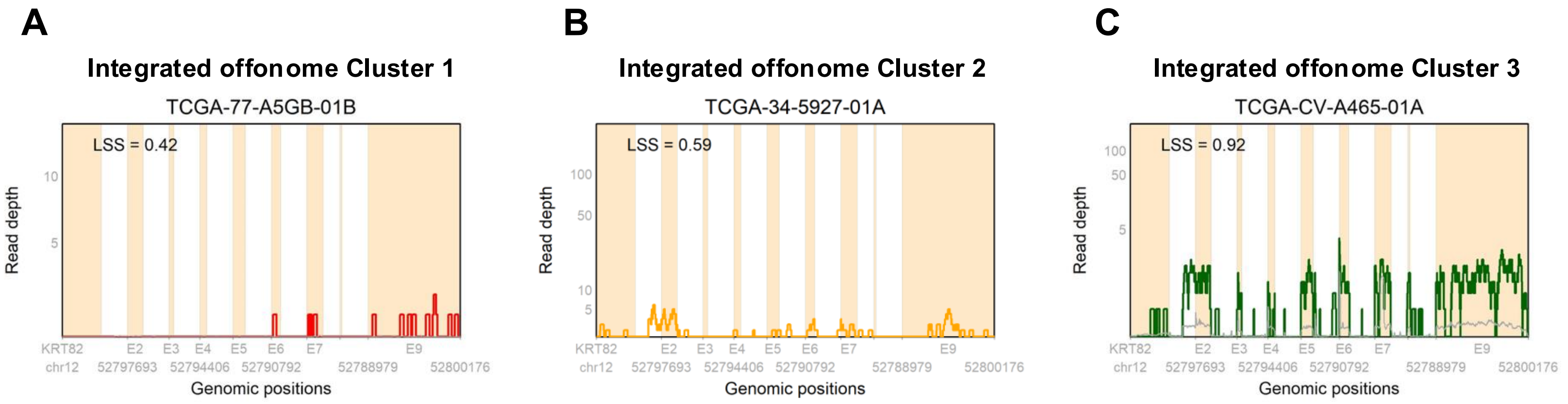

Supplementary Figure 6. Lowly expressed keratinization genes associated with cancer type clustering This figure shows varying LSS values despite all samples exhibiting low read coverage. We employed base resolution RNA-seq pileup data of the *KRT82* gene, which serves as an example for discerning among the three tumor types illustrated in Figure 4B. Within panels (A-C), we randomly chose one sample from each integrated offonome cluster: (A) from Column cluster 1, (B) from Column cluster 2, and (C) from Column cluster 3. These individual samples are depicted in their corresponding pileup panels. In the pileup plots, exon regions of the *KRT82* gene are highlighted by orange boxes labeled E1-E6 along the X-axis, while intron regions are delineated by white boxes. The Y-axis denotes the read depth of each genomic base location, with the grey line in the pileup data indicating the mean value across all samples within each Column cluster.

Supplementary Figure 7. Hierarchical clustering for integrated data using other expression measures

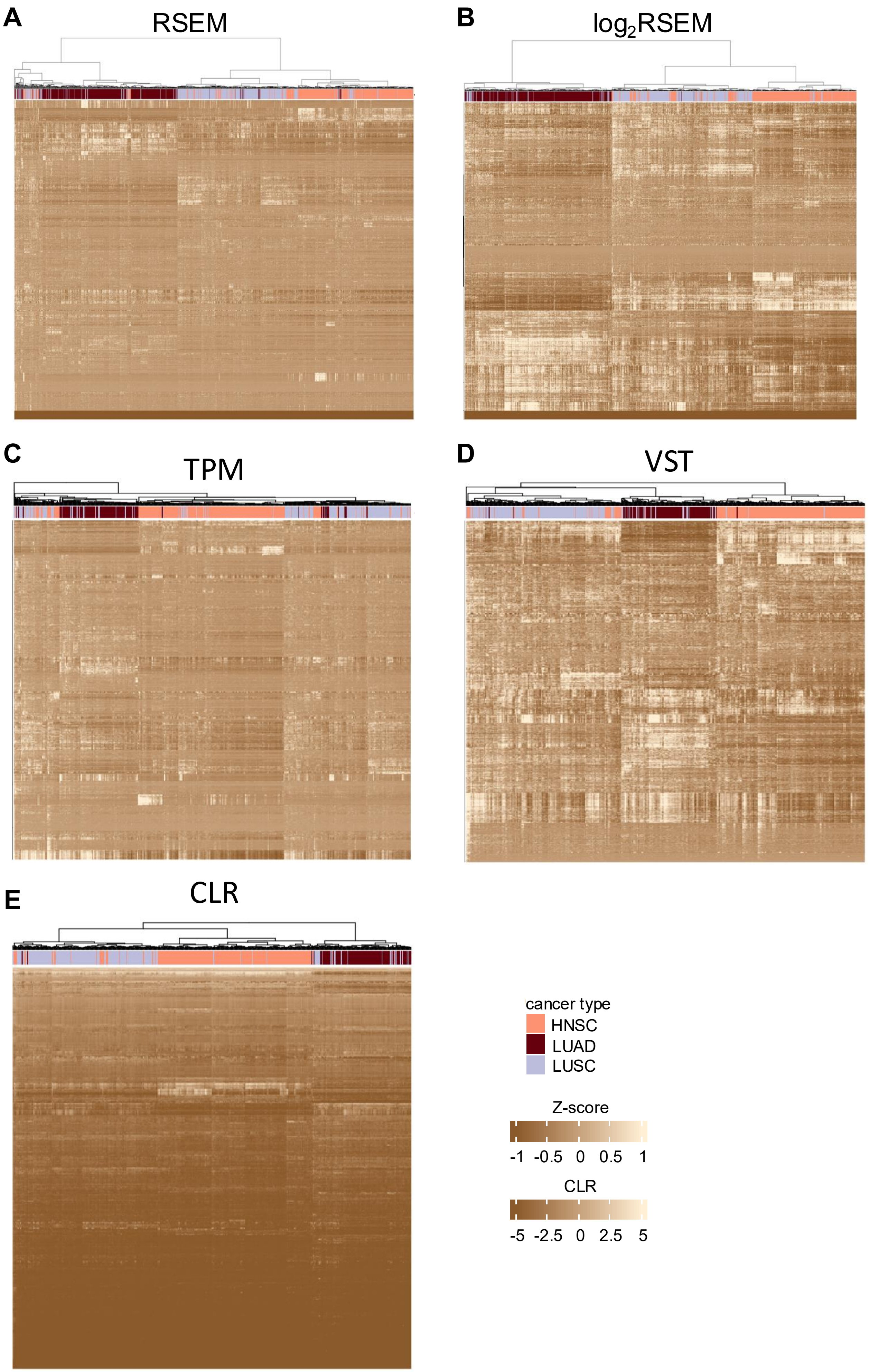

Supplementary Figure 7. Hierarchical clustering for integrated data using other expression measures
Heatmaps of clustering result for integrated offonome (6328 genes) form Figure 4B by using different expression measure which are row scaled (A) RSEM, (B) log_2-transformed RSEM (C) Transcripts Per Million (TPM) and (D) variance stabilizing transformation (VST). In figure A-D, we applied *z*-score transformation for each row. (E) Centered-log ratio measure also utilized in clustering for integrated offonome. Heatmap column annotations shows different tumor types.
